# A novel data mining system points out hidden relationships between immunological markers in multiple sclerosis

**DOI:** 10.1186/1742-4933-10-1

**Published:** 2013-01-10

**Authors:** Maira Gironi, Marina Saresella, Marco Rovaris, Matilde Vaghi, Raffaello Nemni, Mario Clerici, Enzo Grossi

**Affiliations:** 1 INSPE, San Raffaele Hospital, Milan, Italy; 2 Don Carlo Gnocchi Foundation, IRCCS, S. Maria Nascente, Milan, Italy; 3 Semeion Research Center, Via Sersale 117, Rome, 00128, Italy; 4 CAM, Polidiagnostic Center, Monza, Italy

**Keywords:** Multiple Sclerosis, Immunological network, AutoCM method, Biomarkers of inflammation and neurodegeneration

## Abstract

**Background:**

Multiple Sclerosis (MS) is a multi-factorial disease, where a single biomarker unlikely can provide comprehensive information. Moreover, due to the non-linearity of biomarkers, traditional statistic is both unsuitable and underpowered to dissect their relationship. Patients affected with primary (PP=14), secondary (SP=33), benign (BB=26), relapsing-remitting (RR=30) MS, and 42 sex and age matched healthy controls were studied. We performed a depth immune-phenotypic and functional analysis of peripheral blood mononuclear cell (PBMCs) by flow-cytometry. Semantic connectivity maps (AutoCM) were applied to find the natural associations among immunological markers. AutoCM is a special kind of Artificial Neural Network able to find consistent trends and associations among variables. The matrix of connections, visualized through minimum spanning tree, keeps non linear associations among variables and captures connection schemes among clusters.

**Results:**

Complex immunological relationships were shown to be related to different disease courses. Low CD4IL25+ cells level was strongly related (link strength, ls=0.81) to SP MS. This phenotype was also associated to high CD4ROR+ cells levels (ls=0.56). BB MS was related to high CD4+IL13 cell levels (ls=0.90), as well as to high CD14+IL6 cells percentage (ls=0.80). RR MS was strongly (ls=0.87) related to CD4+IL25 high cell levels, as well indirectly to high percentages of CD4+IL13 cells. In this latter strong (ls=0.92) association could be confirmed the induction activity of the former cells (CD4+IL25) on the latter (CD4+IL13). Another interesting topographic data was the isolation of Th9 cells (CD4IL9) from the main part of the immunological network related to MS, suggesting a possible secondary role of this new described cell phenotype in MS disease.

**Conclusions:**

This novel application of non-linear mathematical techniques suggests peculiar immunological signatures for different MS phenotypes. Notably, the immune-network displayed by this new method, rather than a single marker, might be viewed as the right target of immunotherapy. Furthermore, this new statistical technique could be also employed to increase the knowledge of other age-related multifactorial disease in which complex immunological networks play a substantial role.

## Background

Multiple Sclerosis (MS) is an autoreactive Tcell–driven CNS disease representing the most common cause of non-traumatic disability for the young adult. Different clinical phenotypes can be recognized in this disease: the most common clinical phenotype of MS is the relapsing-remitting (RR) form, which is characterized by an acute onset of symptoms and signs suggestive of neurological dysfunction, followed by complete or partial recovery. The long-term prognosis of RR is usually unfavorable, since patients enter the so-called secondary progressive (SP) phase of the disease and accumulate irreversible neurological disability. Approximately 50% of the patients with RR will have a transition to SP by 15 years after disease onset in the absence of any treatment [1]. A different disease pattern, primary progressive (PP) MS, is seen in patients showing a progressive course from the onset; an even rarer form of disease is benign (BB) MS. In this case absent or minimal neurological impairment are present many years after the onset manifestation. Notably, whereas RR MS appears to be largely driven by inflammatory processes, neurodegeneration plays an important role in the chronic brain and spinal cord injury in patients with PP MS and SP MS. Unfortunately, in spite of the new imaging biomarkers discovered, the state and course of the disease remains rather unpredictable. Since long time ago several studies [2,3] tried to finger out whether peculiar immunological signatures could be associated with different courses of MS. Literature data indeed suggest that certain immunological relevant markers could identify specific disease state and disease activity in MS patients [4]. Moreover findings in humans are corroborated by results from experimental autoimmune encephalomyelitis (EAE), a well-established animal model of MS, where a specific pattern of lymphocyte infiltration and cytokine secretion along with the different phases of the disease has been described [5]. Careful analysis of the alterations in immune processes should further advance knowledge of the pathogenic mechanisms of this disease, and might have predictive value toward disease evolution.

Unfortunately, the overall immunological results of literature are sometimes conflicting and often insufficient to disclose the effective relationship among studied variables. Although differences between trial designs, patients’ population, immunological markers, and technical methodologies can explain the most of this inconsistency, statistical analyses might be an important factor to be considered. Thus, traditional statistical algorithms are both unsuitable and underpowered to dissect the relationship between high number of markers due to the non-linearity and complexity of the immunological network; a fuzzy clustering approach based on evolutionary programming (PST) and Semantic connectivity map (AutoCM) could find the natural associations among immunological markers.

This new artificial adaptive system is able to define the strength of the associations of each variable with all the others and to visually show the map of the main connections of the variables and the basic semantic of their ensemble. The matrix of connections, visualized through minimum spanning tree, by AutoCM, keeps non linear associations among variables and captures connection schemes among clusters. These artificial adaptive systems were previously applied to clinical dataset of Alzheimer patients [6] where they successfully disclosed previously unknown connections among the large body of factors related to this multifactorial disease.

In this pilot study we investigated immunological markers' network, using AutoCM in 103 MS patients and 42 healthy controls (HC). The same clinical and immunological dataset was previously studied with traditional statistical analyses [7].

The aim of this study was to verify these data with a completely different statistical technique, and to investigate whether this revolutionary mathematical approach can increase the intelligibility of the immunological connections.

## Results

**Figure 1:**
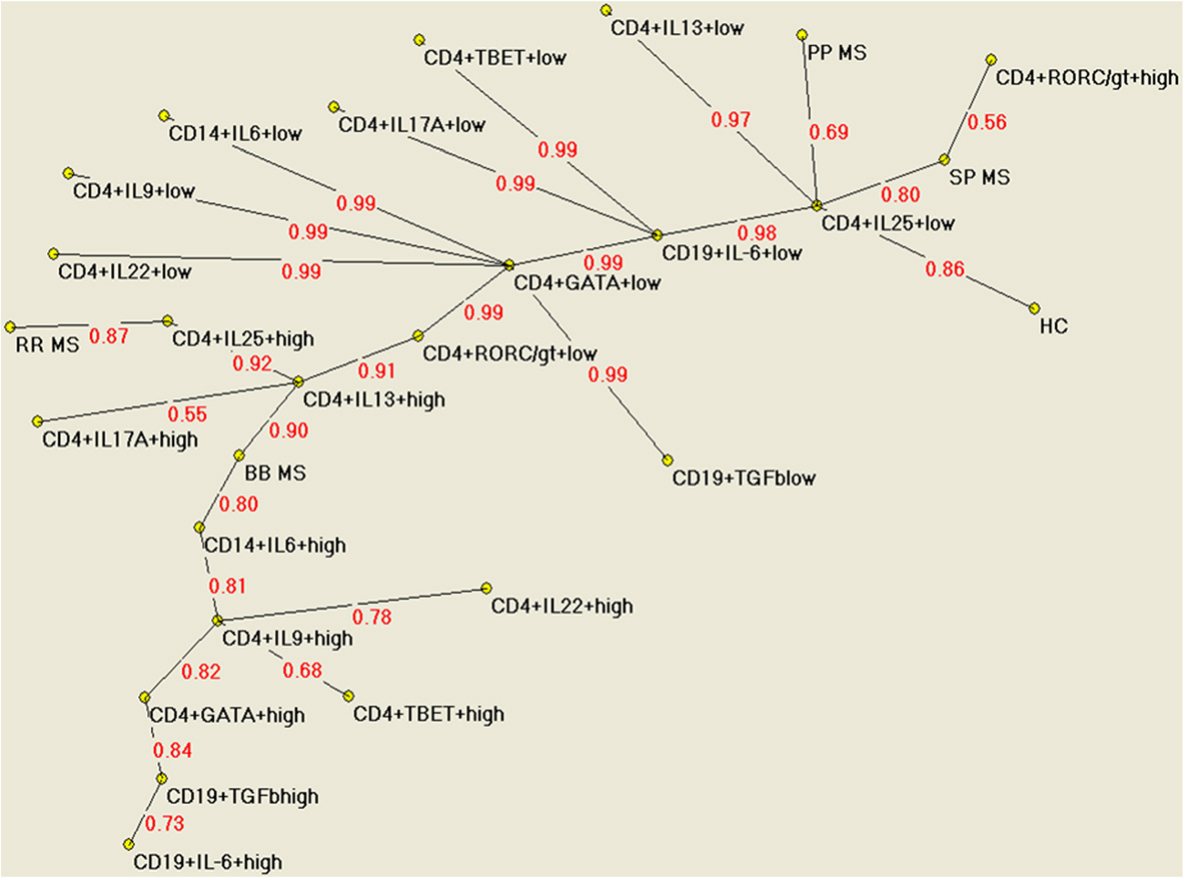
**Semantic connectivity map of the pathway linking immunological markers to different phenotypes of MS. **Semantic connectivity map of variables on study: values ranging from 0 (no association) to 1 (the strongest association) express the strength of association.

The main relationships between immunological markers and MS clinical phenotypes emerging from the AutoCM analyses are shown in Figure 1. Results clearly indicated that the progressive forms (PP and SP MS) of the disease can be visually separated from the other clinical phenotypes (RR and BB). Thus, the progressive forms of MS are associated with different immunological markers compared to RR MS and BB MS. By means of AutoCM, it is possible to obtain not only the direction of the association as provided by standard statistical analyses, but importantly also the strength of this association. A reduced percentage of CD4/IL25+ cells was strongly (ls=0.81) related to secondary progressive MS, this phenotype being also (ls=0.56) associated to increased amounts of CD4/ROR+cells. On the opposite part of the graph, benign phenotypes (BB MS)were clearly related to higher quantities of CD4+/IL13 and CD14+/IL6 cells (ls=0.80). This relationship depicts a comparable impact of anti-inflammatory (CD4+IL13 cells) factors and of inflammatory markers (CD14+IL6 cells) in the shaping of this favorable clinical course. Interestingly, the RR MS form was directly associated with CD4+/IL25 cells (ls=0.87), whereas an indirect association with CD4+/IL13 cells was detected. In turn, CD4+IL25 cells are strongly (ls=0.92) associated to CD4+IL13 cells, strengthening the well known induction activity of the former (CD4+/IL25) on the latter (CD4+/IL13) T lymphocyte populations [8,9]. Another interesting topographic data is the isolation of Th9 cells (CD4IL9) from the main part of the immunological network related to MS, suggesting a possible secondary role of these recently discovered immune cells in MS. 

## Discussion

Immunological markers are objective measures with clinical and scientific relevance. They can be useful in diagnostic definition/exclusion (e.g., anti acquaporin antibodies to differentiate MS from Neuromyelitis Ottica), to assess treatment response (e.g., anti IFN neutralizing antibodies in non-responder patients), but also to dissect pathogenetic mechanisms of disease [10-12]. Due to the complexity and redundancy of immune system, investigating the pattern of correlations among immune markers is a challenging task for traditional statistic. The latter can provide binary correlation, and show immunological pattern best associated to a clinical phenotype but it is unsuitable to finger out the non-linear interconnections among variables. In order to find the natural associations among immunological markers we applied a fuzzy clustering approach based on evolutionary programming (PST) and Semantic connectivity map (AutoCM) to the data of a wide immunological study, dissecting new pathways of immunological mechanisms [7]. AutoCM is a special kind of Artificial Neural Network able to find consistent trends and associations among variables. The matrix of connections, visualized through minimum spanning tree, keeps non linear associations among variables and captures connection schemes among clusters. The use of this mathematical approach, as shown by literature [13] should disclose the complex relationships among markers, otherwise impossible to be fingered out by traditional analyses. A global view can suggest how AutoCM can visually separate the progressive forms of disease (PP and SP) from RR and BB phenotypes, connecting the last to a network of different immunological markers. This finding could be the immunological support of the better prognosis and less destructive mechanisms associated to RR and BB compared to SP and PP.

In particular, AutoCM found a strong relationship between RR and high percentages of IL25-expressing CD4 cells. These lymphocytes are central in down-regulating the inflammatory potential of TH17 cells, directly or via IL-13- secreting CD4 cells [8,14]. Thus, it has been suggested that the disease can enter temporary quiescence phase (remission) by these anti-inflammatory mechanisms. Interestingly the AutoCM caught the sense of this relationship, showing the direct link of RR phenotype to IL-25 CD4 cells and indirect with IL-13- secreting CD4 cells, confirming preliminary data from literature but even adding new information. Our previous study showed that IL-6-expressing CD14+ cells were significantly augmented in all the different MS phenotypes. The differentiation processes of these cells occurring when they get the target tissue, mainly depends on inflammatory or anti-inflammatory milieu they find. Accordingly, most of these cells find in the inflammmated CNS the ideal milieu priming their differentiation into functional myeloid dendritic cells able to support the proliferation and expansion of TH17 cells [15]. In turn these latter cells orchestrate the effective inflammatory damage to myelin and cells. Interestingly, the new finding provided with this analyses suggest that IL-6-expressing CD14+ cells play a role also in benign course, but are strictly associated (in the visual map) with IL-13- expressing CD4 cells. These lymphocytes are endowed with anti-inflammatory properties [16] and therefore could turn off the potential detrimental effect of IL-6-secreting CD14+ cells as well as that of TH17 cells. The clarity and strength of the relationship between IL-6-secreting CD14+ cells and IL-13- secreting CD4 cells definitely comes out better by AutoCM analyses. Considering the upper part of the graph, the most striking element is the central hub represented by low percentage of IL-25/CD4 cells. These cells, as aforementioned, represent one of the most important ways to regulate an inflammatory response [8]. Low levels of IL-25 CD4 cells could be associated to a predisposing condition to inflammation that would be further enhanced by the co-presence of inflammatory cells (e.g., CD4+ROR+cells). The co-existence of low IL-25 CD4 and high CD4+ROR+cells is shown by AutoCM as the signature of SP phenotype. These data suggest that the defect of a counter-regulative (anti-inflammatory) mechanism could be a possible explanation for the particularly aggressive course of SP MS. Figure 1 shows how strong is also the link between low IL-25 CD4 and PP as well as between IL-25CD4 and HC . However it’s worth to note that by AutoCm analyses PP is not shown to be associated with high percentages of CD4+/ROR+ cells as SP is; it is tempting to speculate that this difference might play a role in determining the clinical course of these different phenotypes. Our data are in line with literature that highlights the paucity and controversial role of inflammation present in PP comparing with other MS phenotypes [17]. Notably, low levels of IL-25/CD4 cells were also associated to healthy subjects, but again in this group the association with high inflammatory subsets (CD4+ROR+cells) lacks. This data could suggest that a defective anti-inflammatory response could not be per se a sufficient condition leading to multiple sclerosis. The carriership of other immunological risk factors (beyond the focus of our investigation) and/or non-immunogical (genetic, epigenetic, and environmental) variables can make this anti-inflammatory impairment the trigger for the disease. Interestingly, CD4+IL9 secreting cells (Th9) were clearly “topographically excluded”. This newly described functional immunological phenotype has been suggested to be involved in the pathogenesis of autoimmune diseases [18,19]. Moreover these cells seem to be the trigger of the inflammatory response driven by Th17 during MS [20]. However our previous study [7] failed to detect a clear difference in IL-9-secreting cells between any clinical MS phenotype and HC. AutoCM strengthen this finding with a different analysis indicating the contemporary association of Th9 cells to the 3 main immune CD4+ T lymphocytes phenotypes: Th2 (CD4GATA+), Th1 (CD4TBET) and Th17 (CD4IL22). In conclusion, we presented a new mathematical algorithm to dissect a complex network of variables. The AutoCM system reshapes the distances among immunological variables elsewhere studied by standard approach.

## Conclusion

In conclusion AutoCM clearly show PP and SP strongly associated to “lowCD4IL25”, conversely BB appear related to both “high CD14 IL13” and “high CD4IL6” immunological signature. Being MS a multifactorial disorder, several other biological variables (genetic, epigenetic, transcriptomics…) rely on the clinical differences of these opposite phenotypes. Notwithstanding the need of other reliable biological markers, our findings shed further light on the meaning of the immunological markers associated to different disease courses”

The superiority of AutoCM method compared to those obtained with Euclidean or other linear-correlation-distance-matrix-based analyses is evidenced by the global information it provides. Not only the reciprocal binary connection among biological and clinical variables, but the totality of these connections and the strength of the entire immunological network can be disclosed by this new revolutionary method. These data sound to suggest that future studies based on this new mathematical approach could better define also the relationship between the inflammatory component and neurodegeneration factors involved in MS. Moreover, AutoCM could be a useful tool to finger out complex networks underpinning age-related multifactorial diseases.

Increasing knowledge of so far unveiled relationships of factors involved in these diseases could pave the way for more specific drugs.

## Methods

We performed a depth immune-phenotypic and functional analysis of peripheral blood mononuclear cell (PBMCs) by flow cytometry on 103 MS patients and 42 HC. Studied population consisted of 103 MS patients with relapsing remitting (RR, n= 30), benign (BB, n=26), primary (PP, n=14), or secondary progressive (SP, n=33) MS diagnosed according international consensus criteria [21].

Clinical history and drug assumption were recorded and neurological examination was performed, including the Expanded Disability Status Scale (EDSS) rating. Patients were considered affected by BB MS when EDSS score was ≤ 3.0 and disease duration equal to or longer than 15 years [22]. Median disease duration was 7 years (range: 1–29 years); the median Expanded Disability Status Scale (EDSS) score was 1.5 (range: 1–6). Demographic and clinical data are reported in Table 1. Patients were recruited at Multiple Sclerosis Center Fondazione Don Carlo Gnocchi (Milan) and CAM, Polidiagnostic Center (Monza). 

**Table 1:** Demographic and clinical data of studied patients and healthy controls

	**RR**	**BB**	**SP**	**PP**	**HC**
N	30	26	33	14	42
Age (range)	40 (20–59)	45 (36–61)	49 (32–67)	50 (37–64)	48 (32–62)
F:M	19:11	18:8	21:12	6:8	28:14
Disease duration	7	21	20	12	--
EDSS	1.5	2	6.5	6	--

Relapsing remitting (RR); benign (BB); secondary progressive (SP); and primary progressive (PP) multiple sclerosis patients were selected. A group of 42 sex and age matched healthy controls (HC) were enrolled in the study as well. F: female, M: male. Average and range of age are reported, EDSS: Expanded disability status scale. *Age values are not significantly different among MS subgroups; EDSS, as expected, is higher in PP and SP subgroups in comparison with RR and BB.*

All subjects had to be free of relapse or of a confirmed disease progression in the last 30 days and stopped any immunosuppressive drugs at least 12 months before enrollment; symptomatic drugs (SSRI, Baclofen, Oxibutine) were accepted. Subjects with a clinically significant or unstable medical condition (cardiovascular, pulmonary, hepatic, gastrointestinal, renal, metabolic diseases or malignancies) were excluded from the study. Following acquisition of informed consent, fasting blood samples were collected in the morning and immediately analyzed.

### Blood sample collection and cell separation

Whole blood was collected in vacutainer tubes containing ethylenediaminetetraacetic acid (EDTA) (Becton Dickinson & Co., Rutherford, NJ, USA). Peripheral blood mononuclear cells (PBMC) were separated on lymphocyte separation medium (Organon Teknika Corp., Durham, NC, USA) and washed twice in PBS. Viable leukocytes were determined by Scepter^TM^ Handheld Automated Cell Counter (Millipore, MA, USA).

### Synthesis of the MBP peptides

Thirty-one HLA I restricted and 7 HLA II restricted promiscuous peptides partially overlapping and spanning the whole Myelin Basic Protein (MBP) were synthesized using Fmoc chemistry. Peptides purity, as assayed by HPLC, was > 70%, and their composition was verified by mass spectrometry. Lyophilized peptides were dissolved at 25 mg/ml in DMSO or sterile water to prepare peptide pools (10 mg/ml final concentration).

### Stimulation of PBMC for FACS analysis

1 x 10^6^ PBMC were stimulated with non-immunogenic peptides or with a pool of the MBP peptides (10 mg/ml) + anti-CD28 mAb (clone 37407.111; R&D Systems, Inc., Minneapolis, MN,USA) (1 μg/ml) to facilitate co-stimulation, at 37° C in a humidified 5% CO2 atmosphere for 24 hours. Fr cytokine analyses, 10 μg/ml Brefeldin A (Sigma-Aldrich,St. Louis, MO,USA) was added to the cell cultures during the last 6 h of stimulation to block protein secretion.

### Immunofluorescent staining

PBMC were stained with CD4, CD19 and CD14 mAbs (Beckman Coulter, Brea, CA, USA), washed in PBS and treated with FIX and PERM (FIX & PERM Cell Permeabilization kits; eBioscience San Diego, CA, USA), then fixed for 10 min in fixation medium (100μl), washed, and suspended in 100μl of permeabilization medium with mAbs against the following proteins: anti-RORC/RORγτ, anti-T-bet, anti- GATA3, anti- NFkB, anti-NFATc1, or with IFN-γ IL-4, IL-6, IL-9, IL-10, IL-12, IL-13, IL-17, IL-21, IL-22, IL-23, IL-25 and TGF-β- specific mAb FITC or PE-conjugated for 30 minutes at 4°C in the dark.

### Monoclonal Abs

The following mAbs were used in this study: Phycoerythrin-Cyanin-7 (PC7)- labeled anti-CD4 (clone SFCI12T4D11) (mouse IgG1), Fluorescein Isothiocyanate (FITC)-or Phycoerythrin-Cyanin-5 (PC5) labeled anti-CD14 (clone 116), (Beckman-Coulter, Fullerton, CA). The intracellular molecule detection mAbs used were: anti-human Phycoerythrin (PE)-coupled IL-10 (clone JES9D7; mouse IgG1 isotype; Caltag Laboratories), anti-human IL-4 FITC (clone MP4-25D2, rat IgG1_k_ isotope, eBioscience Cornerstone Court West, San Diego, CA), anti-human IFNγ-PE, anti-human IL-6- FITC (clone 1936, mouse IgG_2B_ isotope, R&D Systems Inc., Minneapolis), anti-human IL-9- PE (clone MH9A4, mouse IgG_2B,k_ isotype, Biolegend, San Diego, CA), anti-human IL-12- FITC (clone 27537, mouse IgG_1_ isotype, R&D), anti-human IL-13 FITC (clone 32007, mouse IgG1isotype, R&D) anti-human IL-17-PC5 (clone BL168, mouse IgG_1k_ isotype, Biolegend), anti-human IL-21-PE (clone 3A3-N2, mouse IgG_1_ isotype, eBioscience), anti-human IL-22-PE (clone 142928, mouse IgG_1_ isotype R&D), anti-human IL-23-PE (clone C11.5, mouse IgG_1k_ isotype, Biolegend), anti-human IL-25-PE (IL-17E, clone 182203, mouse IgG1isotype, R&D) anti-mouse/human RORC/RORγτPE (clone AFKJS-9, rat IgG_2a_ isotype, eBioscience), anti-mouse/human T-bet- PE (clone 39D, mouse IgG_1_ isotype, eBioscience), anti-mouse/human GATA3-PE (clone TWAY, rat IgG_2B,k_ isotype, eBioscience), anti- human NFkB-FITC (clone C-5, mouse IgG_2a_ isotype, Santa Cruz Biotechnology, Santa Cruz, CA), anti- human NFATc1-PE (clone H-10, mouse IgG_1_ isotype, Santa Cruz-Biotechnology).

### Cytometric analysis

Analyses were performed using a Beckman-Coulter Cytomics FC-500 flow cytometer equipped with a single 15 mW argon ion laser operating at 488 nm and interfaced with CXP Software 2.1. Two-hundred-thousands events were acquired and gated on CD4 or CD14 expression and side scatter properties. Data were collected using linear amplifiers for forward and side scatter and logarithmic amplifiers for FL1, FL2, FL4 and FL5. Samples were first run using isotype control or single fluorochrome-stained preparations for color compensation. Rainbow Calibration Particles (Spherotec, Inc. Lake Forest, IL) were used to standardize results in samples obtained over time.

### Mathematical methods

The analysis performed on this database has the aim of increasing our understanding of the complex pathway linking immunological markers to different phenotypes of MS. This goal has been achieved through a new data mining method, based on a particular artificial adaptive system, the Auto Semantic Connectivity Map (AutoCM), that is able to compute the association strength of each variable with all the others in any dataset (i.e. in terms of many-to-many rather than dyadic associations). The architecture and mathematics of AutoCM is described elsewhere [13,23].

In non-technical terms, AutoCM is a new data mining tool based on an Artificial Neural Network developed at Semeion Research Center [24] that is especially effective at highlighting any kind of consistent patterns and/or systematic relationships and hidden trends and associations among variables. Quite uncommonly, the weights determined by AutoCM after the training phase, admit a direct interpretation. Specifically, they are proportional to the strength of many-to-many associations across all variables. This allows a further, useful processing: association strengths may be easily visualized by transforming weights into physical distances. Such a 'translation' proceeds in an intuitive way: couples of variables whose connection weights are higher get relatively nearer, and vice versa. By applying a simple mathematical filter such as the minimum spanning tree to the matrix of distances, a graph is generated, whose use has been already tested in the medical field [6,25], and that is termed connectivity map as detailed by Buscema and colleagues [13,25]. This representation then allows a very intuitive visual mapping of the complex web of connection schemes among variables, and greatly eases the detection of the variables that play a key role in the schemes, i.e. that turn out to be “hubs” of the graph. The system provides also a quantification of the “strength” of links among variables (nodes of the graph) by a numerical coefficient (link strength, ls) ranging from zero to 1.

The value superimposed to the link is proportional to the strength of the link. The strength of the link (ls) ranges from 0 (minimum strength) to 1 (maximal strength).

The strength of the link can be read as the probability of transition from any state-variable to anyone else.

The AutoCM matrix of connections preserves non linear associations among variables, while at the same time capturing elusive connection schemes among clusters that are often overlooked by traditional cluster analyses, and highlighting complex similarities among variables on various dimensions–role, connectivity, essentiality, and so on. The AutoCM algorithms used for all the computations presented in this paper are implemented only by a Semeion proprietary research software, which is exclusively available for academic purposes.

The immunological findings obtained by FACS were translated in absolute natural values, scaled from 1 to zero, becoming single inputs for AutoCM

We transformed the 11 immunological variables in 22 input variables constructing for each of the variable, scaled from zero to 1, its complement (Table 2). 

**Table 2:** Variables' transformation

	**Original variables**	**Variables’ transformation**
1	CD4+RORC/γτ+	1- CD4+RORC/γτ+
2	CD4+IL17A+	1- CD4+IL17A+
3	CD4+IL22+	1- CD4+IL22+
4	CD4+TBET+	1- CD4+TBET+
5	CD4+IL9+	1- CD4+IL9+
6	CD4+GATA+	1- CD4+GATA+
7	CD4+IL13+	1- CD4+IL13+
8	CD4+IL25+	1- CD4+IL25+
9	CD14+IL6+	1- CD14+IL6+
10	CD19+IL-6+	1- CD19+IL-6+
11	CD19+TGFβ	1- CD19+TGFβ

We transformed the 11 immunological variables in 22 input variables and we made for each of the variable, scaled from 0 to 1, its complement as better detailed in the text.

Consider for example the variable CD14_IL6. Absolute natural values range from 0.1 to 60. According to the transformation, 60 (the highest value) becomes 1 and 0.1 (the lowest value) becomes 0. All other natural values are scaled in this new range. For instance: the value 25 becomes 0.41; the value 11 becomes 0.18, etc. The projection of the variable in the map will point out the fuzzy position of CD14_IL6 according to its high values. In the complement transformation, by subtracting the scaled value from 1 (e.g. 11 becomes now 0.82) we allow the system to project and point out the fuzzy position of CD14_IL6 according to its low values. This is important because in non linear systems, the position of high and low values of a given variable is not necessarily symmetric.

In this way the projection of the original variables tended to show high values while the complement transformation tended to show low values of the original variables. In the map we have named these two different forms as high and low. This pre-processing scaling is necessary to make possible a proportional comparison among all the variables and understand the existing links of each variable when the values tend to be high or low.

## Abbreviations

MS: Multiple Sclerosis; HC: Healthy Controls; RR: Relapsing Remitting; SP: Secondary Progressive; PP: Primary Progressive; BB: Benign; AutoCM: Semantic Connectivity Map; EDSS: Expanded Disability Scale; PBMC: Peripheral Blood Mononuclear Cell.

## Competing interests

The author has declared that no competing interest exists.

## Authors’ contributions

MG conceived the study, its design and coordination, critically revised the clinical- immunological and statistical analyses and wrote the manuscript. MS carried out all the immune-phenotypic and functional analysis of PBMCs by flow-cytometry. MR enrolled the patients, critically commented the data and revised the manuscript. MV participated in the critical review of the data and of the manuscript. RN enrolled the patients and critically revised the manuscript. MC participated in the critical review of the immunological analyses and statistical analyses, revised the manuscript and participated in the design of the study. EG carried out all the statistical analyses, critically revised the data, the relationship between clinical and immunological findings and definitely revised the manuscript. We are greatful to Dott Roberto Furlan for his excellent “skeptism” in data revision. All authors read and approved the final manuscript.
